# Comparison of small biopsy specimens and surgical specimens for the detection of EGFR mutations and EML4-ALK in non-small-cell lung cancer

**DOI:** 10.18632/oncotarget.10011

**Published:** 2016-06-14

**Authors:** DeSheng Xiao, Can Lu, Wei Zhu, QiuYan He, Yong Li, ChunYan Fu, JianHua Zhou, Shuang Liu, YongGuang Tao

**Affiliations:** ^1^ Department of Pathology, Xiangya Hospital, Central South University, Changsha, Hunan 410078 China; ^2^ Department of Pathology, School of Basic Medicine, Central South University, Changsha, Hunan 410078 China; ^3^ Cancer Research Institute, School of Basic Medicine, Central South University, Changsha, Hunan, 410078 China; ^4^ Key Laboratory of Carcinogenesis and Cancer Invasion (Central South University), Ministry of Education, Hunan, 410078 China; ^5^ Key Laboratory of Carcinogenesis (Central South University), Ministry of Health, Hunan, 410078 China; ^6^ Center for Medicine Research, Xiangya Hospital, Central South University, Changsha, Hunan, 410008 China

**Keywords:** EGFR, EML4-ALK, small biopsy specimens, surgical specimens, non-small-cell lung carcinoma

## Abstract

Epidermal growth factor receptor (EGFR) mutations and anaplastic lymphoma kinase (ALK) fusion genes represent novel oncogenes that are associated with non–small-cell lung cancers (NSCLC). The feasibility of detecting EGFR mutations and ALK fusion genes in small biopsy specimens or surgical specimens was determined. Of the 721 NSCLC patients, a total of 305 cases were positive for EGFR mutations (42.3%). The rate of EGFR mutations in women was significantly higher than that in men. Histologically, the EGFR mutation rate in adenocarcinomas was significantly higher than that in squamous cell carcinomas. No difference in the EGFR mutation rate was observed between surgical specimens (42.1%) and small biopsy specimens (42.4%), which indicated that the EGFR mutation ratios in surgical specimens and small biopsy specimens were not different. In 385 NSCLC patients, 26 cases were positive for EML4-ALK (6.8%). However, 11.7% of the surgical specimens were EML4-ALK-positive, whereas the positive proportion in the small biopsy specimens was only 4.7%, which indicated that EML4-ALK-positive rate in the surgical specimens was significantly higher than that in the small biopsy specimens. Detection of EGFR gene mutations was feasible in small biopsy specimens, and screening for EML4-ALK expression in small biopsy specimens can be used to guide clinical treatments.

## INTRODUCTION

The global cancer burden is growing at an alarming rate, which drives an urgent need for the implementation of effective prevention strategies. Lung cancer is one of the most critical types [1]. The 5-year relative survival rate of lung cancer patients is gradually improving due to improvements in treatment. In China, lung cancer is the most common incident cancer and the leading of cancer death [2]. Lung cancer is classified by histological criteria into non-small cell lung carcinoma (NSCLC), which consists of three main subtypes (adenocarcinoma, squamous cell carcinoma, and large cell carcinoma), and small cell lung carcinoma. Rare subtypes include glandular tumors, carcinoid tumors, and undifferentiated carcinomas.

Epidermal growth factor receptor (EGFR) mutations are the first druggable targets discovered in NSCLC [3]. Two classes of EGFR mutations, exon 19 deletions and exon 21 substitutions, account for the majority of the EGFR mutations reported (~90%). These mutations are correlated with better responses to gefitinib, erlotinib and afatinib [4–6]. Furthermore, these mutations are more frequently observed in Asian populations, never-smokers, females, and patients with adenocarcinoma [7]. An inverse relationship between cumulative smoking pack-years and the frequency of EGFR mutations has widely been reported [8], which suggests that smoking status has some predictive value for the presence of EGFR mutations. However, the association between source of specimen and EGFR mutations remains controversial, and few data are available regarding the predictive value of the source in specimens for EGFR mutations.

The fusion of the Echinoderm microtubule-associated protein like-4 (EML4) and anaplastic lymphoma kinase (ALK) represents another distinct type of mutation that may drive the development of NSCLC. The fusion protein is highly oncogenic both *in vitro* and *in vivo* and results in the constitutive activation of the ALK pathway and ultimately cancer development [9]. Several clinical trials have demonstrated the remarkable efficacy of crizotinib for the treatment of metastatic NSCLC in patients who harbor ALK rearrangements. These results led to the approval of this agent by the US Food and Drug Administration and the European Medicines Agency [10–13]. EML4-ALK rearrangements were reported to be primarily associated with younger age at diagnosis and with adenocarcinoma [14]. However, the impact of the source of specimens in predicting EML4-ALK rearrangements has not been established.

One of the most challenging problems in clinical practice is the acquisition of adequate tumor tissues for analysis. Therefore, the use of available clinicopathological data to predict the likelihood of certain genetic aberrations is of special significance. Furthermore, EML4-ALK rearrangements and EGFR mutations represent two distinct oncogenic mechanisms that might have different clinicopathological features. With ongoing improvements in modern medical technology, many types of biopsies are widely used in clinic. A small biopsy involves taking a small piece of diseased tissue for pathologic examination for the purposes of obtaining a preoperative diagnosis and optimizing the corresponding treatment. The means for this type of sampling include bronchial fiberscopic techniques, ultrasonic bronchoscopy or percutaneous lung biopsy [15, 16]. The advantages of small biopsies are that they are minimally invasive, simple operations with fewer complications and greater patient acceptance that can generally be performed as outpatient procedures. Furthermore, diagnosis is possible in the vast majority of cases. These techniques have wide clinical uses. Therefore, many small biopsy specimens are used to detect gene mutations. Compared with surgical specimens, the small biopsy specimens may have a low proportion, smaller numbers, and a scattered distribution of the tumor cells. In contrast to histopathologic diagnosis, IHC and genetic testing results, it is difficult to enrich the tumor cells. For small biopsy specimens, simple and rapid detection methods with high sensitivity and strong specificity are required. Here, we detected EGFR mutations using the amplification refractory mutation system (ARMS) in 721 NSCLC patients and measured EML4-ALK expression with the VENTANA ALK (D5F3) immunohistochemistry (IHC) method in 385 NSCLC patients to determine the feasibility of using small biopsy specimens for genetic testing and immunohistochemical detection.

## RESULTS

### ARMS analysis detected EGFR mutations in 721 NSCLC cases

Samples from a total of 721 NSCLC patients evaluated for EGFR mutations. The mean age of the subjects was 65 years (ranging from 16 to 85). Of these patients, 437 (60.6%) were male and 284 (39.4%) were female. Histologically, 583 (80.8%) of the specimens were lung adenocarcinomas, 119 (16.5%) were squamous cell carcinomas, 10 (1.4%) were adenosquamous carcinomas, 5 (0.7%) were large cell carcinomas, and 4 (0.6%) were non-small cell lung cancers (unspecified type).

**Figure 1:**
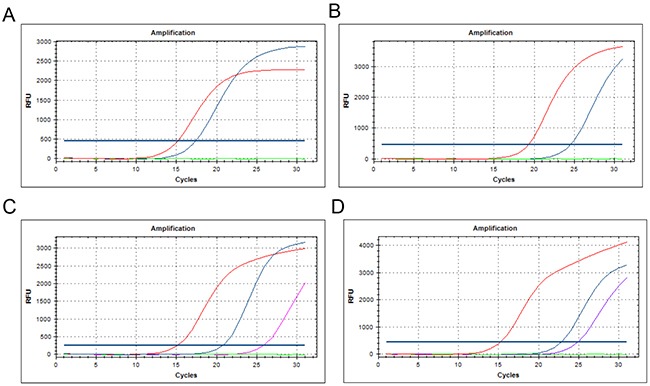
The ARMS method was used to detect EGFR gene mutations including the exon 19 deletion A exon 21 L858R point mutation **B.** the combination of the 19-del and L858R mutation **C.** and L858R and T790M mutations **D.**

EGFR mutations were detected in a total of 305 patients (42.3%): the identified mutations included 155 cases of 19-del (50.8%, Figure 1A), 118 cases of L858R mutations (38.7%, Figure 1B), 4 cases of L861Q mutations (1.3%), 6 cases of G719X mutations (2.0%), 2 cases of S768I mutations (0.7%), and 7 cases of 20-in (2.0%). Single mutations in exons 19 and 21 accounted for 90.8% of all mutations, and single mutations in exons 18 and 20 accounted for 4.9% of all mutations. In addition, 13 cases with double locus mutations, including 3 cases with both 19-del and L858R (Figure 1C), 6 cases with both L858R and T790M (Figure 1D), 2 cases with both 19-del and L861Q, 1 case with both G719X and L861Q, and 1 case with L858R and an exon 20 insertion mutation.

### The correlation between EGFR gene mutations and the lung cancer clinical pathological indicators

EGFR mutation testing results showed that the EGFR mutation rate in the female patients was 59.5% (169/284), which was significantly higher (P<0.01) than that of male patients (31.1%, 136/437). With respect to the histological types, the EGFR mutation rate among the lung adenocarcinomas (46.9%), was significantly higher than that observed in the squamous cell carcinomas (10.1%) (P<0.01) (Table 1).

**Table 1:** The correlation between EGFR gene mutation and lung cancer clinical pathological indicators

Group	Total cases	Mutation cases	Mutation rate	P
Male	437	136	31.1%	0.000
Female	284	169	59.5%	
Adenocarcinoma	584	274	46.9%	0.000
Squamous carcinoma	109	11	10.1%	

### The comparison of EGFR mutation rate between small biopsy and surgical specimens for different clinical pathological indicators

Among the 721 NSCLC patients, the EGFR mutation rate detected in the surgical specimens (42.1%) was not significantly different from that of the small biopsy specimens (42.4%) (P > 0.05) (Table 2). With respect to gender and the histological types, the differences between the EGFR mutation rates for the small biopsy and surgical specimens was not statistically significant (P > 0.05). Furthermore, the differences in the 19-Del and L858R mutation rates between the small biopsy and surgical specimens were also not statistically significant (P > 0.05) (Table 3).

**Table 2:** EGFR mutation rate analysis in 721 NSCLC patients

	Total cases	mutation cases	mutation rate	P
Surgical specimens	171	72	42.1%	0.952
Small biopsy specimens	550	233	42.4%	
Total	721	305	42.3%	

**Table 3:** Comparison of the EGFR mutation rates for small biopsy and surgical specimens

		Total cases	mutation cases	mutation rate	P
Male	Surgical specimens	112	33	29.5%	0.661
	Small biopsy specimens	325	103	31.7%	
Female	Surgical specimens	59	39	66.1%	0.246
	Small biopsy specimens	225	130	57.8%	
Adenocarcinoma	Surgical specimens	139	70	50.4%	0.787
	Small biopsy specimens	416	204	49%	
Squamous carcinoma	Surgical specimens	23	2	9.4%	0.920
	Small biopsy specimens	96	9	10.4%	
19-Del	Surgical specimens	171	40	23.4%	0.490
	Small biopsy specimens	550	115	20.9%	
L858R	Surgical specimens	171	30	17.5%	0.634
	Small biopsy specimens	550	88	16.0%	

### The comparison of EML4 - ALK positive rate between small biopsy and surgical specimens

**Figure 2:**
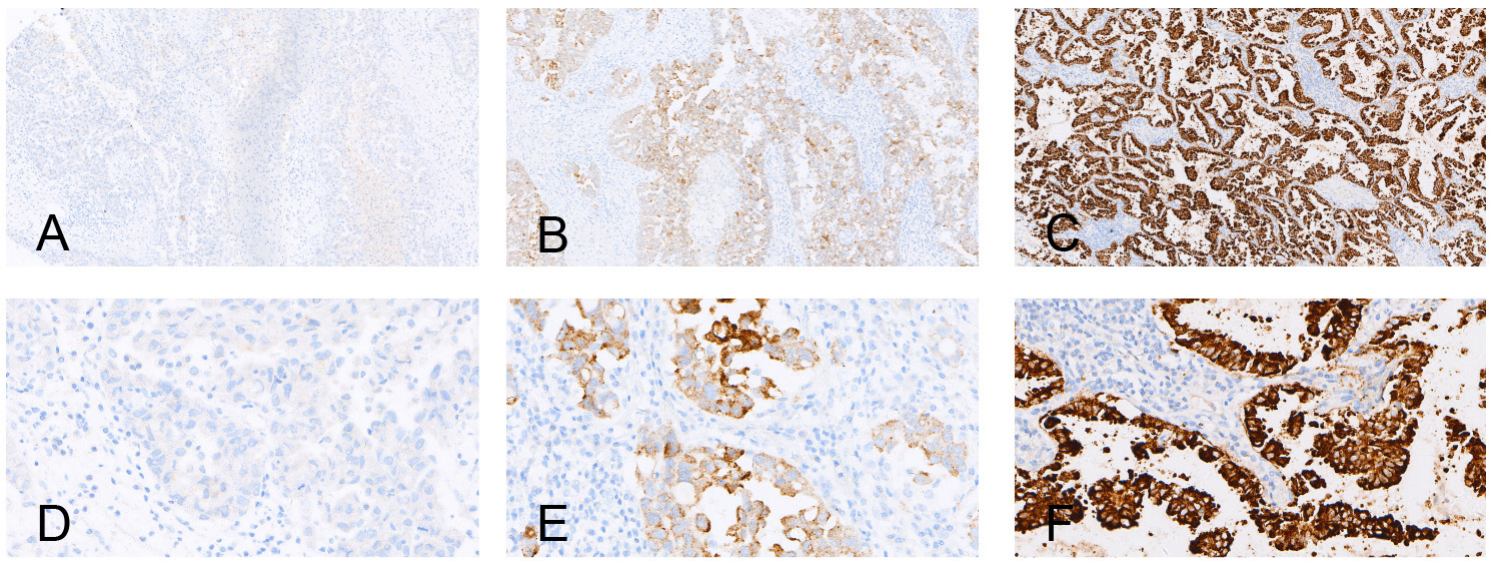
Examples of the immunohistochemical analysis of EML4-ALK **A, B**, and **C.** show the negative control, positive control and an EML4-ALK (+) case (strong granular cytoplasmic staining) (×100), respectively; **D.** **E.** and **F.** show the negative control, positive control and an EML4-ALK (+) case (strong granular cytoplasmic staining) (×400), respectively.

A total of 385 NSCLC patients were recruited for the detection of EML4-ALK. The mean age of these subjects was 63 years, with a range of 17 to 83. Of these patients, 240 (62.3%) were male and 145 (37.7%) were female. Histologically, 293 (76.1%) of the specimens were lung adenocarcinomas and 92 (23.9%) were squamous cell carcinomas. We detected 26 EML4-ALK-positive cases (Figure 2) for a total positive rate of 6.8%. Of these positive cases, 13 were identified in surgical specimens (11.7%) whereas 13 of the small biopsy specimens were positive (4.7%). The EML4-ALK positive rate of the surgical specimens was thus significantly higher than that of the small biopsy specimens (P < 0.05). With respect to gender and the histological types, the EML4-ALK positive rate of the surgical specimens was also significantly higher than the small biopsy specimens (P < 0.05) (Table 4)

**Table 4:** The comparison of the EML4–ALK-positive rates for small biopsy and surgical specimens

		Total cases	Positive cases	positive incidence	P
Total cases	Surgical specimens	111	13	11.7%	0.014
	small biopsy specimens	274	13	4.7%	
Male	Surgical specimens	74	8	10.8%	0.002
	small biopsy specimens	166	3	1.8%	
Female	Surgical specimens	37	5	13.5%	0.463
	small biopsy specimens	108	10	9.3%	
Adenocarcinoma	Surgical specimens	85	10	11.8%	0.205
	small biopsy specimens	208	15	7.2%	
Squamous carcinoma	Surgical specimens	22	1	4.5%	0.073
	small biopsy specimens	70	0	0	

## DISCUSSION

In clinical practice, the discrimination of EGFR mutations and ALK rearrangements in NSCLC has critical therapeutic implications. EGFR mutations confer sensitivity to EGFR tyrosine kinase inhibitors (TKIs) whereas patients with ALK rearrangements response well to ALK TKIs. However, ALK rearrangements are associated with resistance to EGFR TKIs. Because some features including adenocarcinomoid histology and sample source are common to both EML4-ALK rearrangements and EGFR mutations, it is important to investigate other distinct features of these two genetic aberrations from the same samples. To our knowledge, this is the first study to investigate the relationship between the clinicopathological features and the presence of EML4-ALK rearrangements and EGFR mutations in the small biopsy specimens.

In the context of a significantly higher incidence of lung cancer in recent years, targeted molecular therapy has become an important treatment approach for patients with lung cancer. Among these targeted therapeutic agents, TKI based on the EGFR mutations have been widely used in the clinic, and the ability to provide information on the EGFR mutations in clinical tumor tissues with the goal of guiding targeted therapy in a timely manner has become an important goal of lung cancer molecular detection [17–20]. However, approximately 70-85% of new lung cancers are not suitable for surgical therapy, which means that only small biopsy specimens that can be obtained by puncture or bronchial microscopic biopsy are available for diagnosis or further genetic testing [21].

We use the ARMS method to detect EGFR mutations in the primary NSCL cases. In these samples, 305 positive mutations were detected, with 19-Del and L858R mutations being the most common. Some rare mutations and double locus mutations were also observed. The results showed that the EGFR mutation rate in the women was significantly higher than in the men. With respect to the histological type, the EGFR mutation rate in adenocarcinomas was significantly higher than that in squamous cell carcinomas, which is consistent with other reports. We also found no significant difference between the observed EGFR mutation rates in the surgical specimens and small biopsy specimens. More specifically, there were no significant differences between the small biopsy and surgical specimens with respect to the 19-Del and L858R mutation rates. The findings suggest that the specificity and sensitivity for the detection of EGFR gene mutations are consistent between the small biopsy specimens and surgical specimens. Furthermore, the use of small biopsy specimens for EGFR gene mutation detection is completely feasible.

The anaplastic lymphoma kinase (EML4-ALK) fusion gene is an important biological marker of NSCLC. EML4 and ALK are two genes is located in p21 and p23 of human chromosome 2, approximately 10 MB apart. Inversion fusion of the two genes can produce the new fusion protein, EML4-ALK [22, 23]. The identification of EML4-ALK-positive tumors is very important for NSCLC patients because ALK tyrosine kinase inhibitors may shrink tumors that are EML4-ALK positive. Crizotinib, an oral ALK inhibitor, has been shown to be an important inhibitor of tumor growth and survival that reduces or slows tumor growth [12, 24]. The identification of new methods for the identification of gene mutations in tumors is important in the development of targeted therapeutic agents.

Currently, the principal detection methods for EML4-ALK include FISH, RT-PCR and IHC [25–29]. Because manual performance of the FISH technique is difficult, time consuming, difficult to standardize and requires fluorescence microscopy, it is not widely used for EML4-ALK screening. Furthermore, RT-PCR is also not recommended for the detection of EML4-ALK rearrangements because there are subtypes of the EML4-ALK rearrangements, and RT-PCR can only detect known mutations. In addition, there are more stringent requirements for the sample preparation due to the rapid degradation of RNA. The IHC method using Roche/VENTANA ALK IHC (D5F3) detection system is the easiest to perform. This technique uses a kit containing high sensitivity/specific ALK antibodies (D5F3) with enhanced second antibody and amplification reagents. Detection of the EML4-ALK protein was tested in the BenchMark series fully automatic immunohistochemical instrument, and the test results were reported as a simple binary score (negative/positive) [30, 31]. We showed that the EML4-ALK-positive rate for the surgical specimens was significantly higher than that for the small biopsy specimens. Independent of the gender and histological types, the EML4-ALK positive rate of surgical specimens was significantly higher than that of the small biopsy specimens. Clearly, the use of small biopsy specimens had an effect on the EML4-ALK positive rate on the basis that they provided smaller quantities of tissue protein. The expression of EML4-ALK was detected by using Roche/VENTANA ALK IHC detection system from the protein expression level and the sensitivity is lower than that from the DNA expression. Moreover, more tumor tissues are needed to get a satisfactory result from the DNA expression level. Therefore, detection of EML4-ALK from small biopsy specimens is better than that from surgical specimens.

In summary, we showed that the use of small biopsy specimens for the detection of EGFR gene mutations was highly feasible. Immunohistochemical analysis was a more suitable method for the detection of EML4-ALK in surgical specimens than in small biopsy specimens. Furthermore, it is a good reference index by screening EML4-ALK expression in small biopsy specimens to guide clinical medicine. The results should improve the assessment of the likelihood of these two genetic aberrations based on available clinicopathological features as well as the understanding of the biological implications of different oncogenic mutations as evaluated in small biopsy specimens.

## MATERIALS AND METHODS

### Ethics, consent and permissions

Approval to review, analyze, and publish the data in this study was given by the Ethics Board of Xiangya Hospital of Central South University. Written informed consent for the collection of medical information was obtained from all patients at their first visit.

### Clinical data

We collected 721 primary NSCLC specimens from the Xiangya Hospital of Central South University between January 2015 and December 2015 for the detection of EGFR gene mutations. The specimens included 171 surgical specimens and 550 small biopsy specimens. The small biopsy specimens included 281 cases of biopsies collected using CT-guided percutaneous lung puncture, 226 bronchoscopy tissues, 28 samples of exfoliated cell pellets from pleural effusions, and 15 lymph node-puncture tissues. All specimens were obtained before treatment, fixed with 4% neutral formaldehyde, and embedded in paraffin.

We collected 385 primary NSCLC specimens at the Xiangya Hospital of Central South University between January 2015 and December 2015 for VENTANA ALK (D5F3) immunohistochemical detection. These samples included 111 surgical specimens and 274 small biopsy specimens. The small biopsy specimens included 153 biopsies collected using CT-guided percutaneous lung puncture, 102 bronchoscopy tissues, 14 samples of exfoliated cell pellets from pleural effusions, and 5 lymph node-puncture tissues. All specimens were obtained before treatment, fixed with 4% neutral formaldehyde, and embedded in paraffin.

### Reagents

The AmoyDx DNA extraction kit and human EGFR gene mutation detection kit (PCR fluorescence probe method) were purchased from Amoy Diagnostics Co Ltd, Xiamen, China; the anti-ALK (D5F3) rabbit monoclonal antibody, Ventana OptiView signal amplification kit, OptiViewIHC detection kits, rabbit monoclonal negative quality control antibody and quality control slice were obtained from Roche Diagnostics Product Co. LTD, Tucson, U.S.A.

### DNA extraction

A total of 10 pieces 5-8 microns thick were sectioned from wax blocks selected from those with more tumor tissues, and 75% alcohol was used to sterilize the equipment between each case to prevent cross contamination. The tumor tissue genomic DNA was extracted using an AmoyDx DNA extraction kit. After the extraction of the DNA, its concentration and quality were evaluated using a Nanodrop ultramicro spectrophotometer, with a quality criterion of 1.7 < OD260 / OD280 < 2.1.

### ARMS analysis

Mutational analysis of the EGFR was carried out according to the ARMS method using a human EGFR gene mutation detection kit (PCR fluorescence probe method) from Amoy Diagnostics Company [32–34]. We detected 29 mutations in 4 exons of the EGFR gene using this procedure. These included three point mutations of G719X in exon 18; 19 deletion mutations in exon 19; T790M, S768I and 3 insertion mutations in exon 20; and L858R and L861Q point mutations in exon 21. The E GFR mutations were detect ed using CFX96-type fluorescent quantitative PCR from Bio-Rad company. Each batch of reactions was set up to include simultaneous positive and negative controls. After the reaction, the fluorescent signal curves and the threshold line were used to interpret the mutation results.

### VENTANA immunohistochemical analysis

After 3 mm thick paraffin slices were baked, the slices were dyed directly using the Benchmark XT automatic immunohistochemical dyeing machine (American VENTANA company). The dyeing procedure was performed according to the instructions provided with the kit. The test results were evaluated using light microscopy. A binary method of interpretation was used as follows: strong granular cytoplasmic staining (any percentage) in the tumor cells was scored EML4-ALK (+), otherwise they were scored EML4-ALK (−).

### Statistical analysis

The chi-square test (or Fisher exact test) and independent samples test were applied to explore the univariate association between the clinicopathological variables and the specific genetic aberrations for the categorical and continuous data for EGFR and EML4-ALK, respectively. All statistical calculations were performed using SPSS version 19.0 (SPSS, Inc., Chicago, IL.) A two-tailed P value of 0.05 was considered significant.
